# Contamination of the Conchos River in Mexico: Does It Pose a Health Risk to Local Residents?

**DOI:** 10.3390/ijerph7052071

**Published:** 2010-05-04

**Authors:** Hector Rubio-Arias, César Quintana, Jorge Jimenez-Castro, Ray Quintana, Melida Gutierrez

**Affiliations:** 1 College of Zoo-technology and Ecology, Autonomous University of Chihuahua, Mozart 3109, Chihuahua 21203, Mexico; E-Mails: equintan@uach.mx (C.Q.); jajimenez@uach.mx (J.J.-C.); rquintan@uach.mx (R.Q.); 2 Department of Geography, Geology and Planning, Missouri State University, 901 S. National, Springfield, MO 65897, USA; E-Mail: mgutierrez@missouristate.edu

**Keywords:** water contamination, Chihuahua, Mexico, metals

## Abstract

Presently, water contamination issues are of great concern worldwide. Mexico has not escaped this environmental problem, which negatively affects aquifers, water bodies and biodiversity; but most of all, public health. The objective was to determine the level of water contamination in six tributaries of the Conchos River and to relate their levels to human health risks. Bimonthly samples were obtained from each location during 2005 and 2006. Physical-chemical variables (temperature, pH, electrical conductivity (EC), Total solids and total nitrogen) as well as heavy metals (As, Cr, Cu, Fe, Mn, Ni, V, Zn, and Li) were determined. The statistical analysis considered yearly, monthly, and location effects, and their interactions. Temperatures differed only as a function of the sampling month (P < 0.001) and the pH was different for years (P = 0.006), months (P < 0.001) and the interaction years x months (P = 0.018). The EC was different for each location (P < 0.001), total solids did not change and total nitrogen was different for years (P < 0.001), months (P < 0.001) and the interaction years x months (P < 0.001). The As concentration was different for months (P = 0.008) and the highest concentration was detected in February samples with 0.11 mg L^−1^. The Cr was different for months (P < 0.001) and the interaction years x months (P < 0.001), noting the highest value of 0.25 mg L^−1^. The Cu, Fe, Mn, Va and Zn were different for years, months, and their interaction. The highest value of Cu was 2.50 mg L^−1^; for Fe, it was 16.36 mg L^−1^; for Mn it was 1.66 mg L^−1^; V was 0.55 mg L^−1^; and Zn was 0.53 mg L^−1^. For Ni, there were differences for years (P = 0.030), months (P < 0.001), and locations (P = 0.050), with the highest Ni value being 0.47 mg L^−1^. The Li level was the same for sampling month (P < 0.001). This information can help prevent potential health risks in the communities established along the river watershed who use this natural resource for swimming and fishing. Some of the contaminant concentrations found varied from year to year, from month to month and from location to location which necessitated a continued monitoring process to determine under which conditions the concentrations of toxic elements surpass existing norms for natural waters.

## 1. Introduction

In the new millennium, water contamination is considered a prominent factor in relation to human health. Mexico has not escaped this phenomenon. Specifically, the Conchos watershed in Chihuahua, servicing more than a million human inhabitants, has been contaminated with arsenic [1], nitrogenous compounds [2] and other elements like Co, Ni and Zn [3]. Some of these parameters, like the arsenic, epitomize a potential challenge to human health. The arsenic has been considered a carcinogen [4,5] and extensive research was conducted worldwide during the last century concerning this element [6–8]. Previously, in 1958, the World Health Organization had established 0.20 mg L^−^^1^ as the International Standard for Drinking Water. It was then changed to 0.05 mg L^−^^1^ in 1963 and after 1993 the considered value was 0.01 mg L^−^^1^ [9]. The Mexican Norm has a Maximum Permitted Value of 0.025 mg L^−^^1^ [10].

Chromium and heavy metals like Cu, Fe, Mn, Ni, V, Zn and Li are other elements which pose potential health concerns when detected in drinking water and in different aquatic environments. Chromium is considered an essential element [11] but can be toxic at some levels and may be a precursor of different diseases [12]. Excessive copper ingestion may cause *short-term acute* symptoms such as diarrhea but long-term effects may cause liver or kidney damage, anemia [13,14], or may even be related to some neurodegenerative conditions such as Alzheimer’s disease [15,16]. Fe is considered essential in a wide range from 3 mg L^−^^1^ to 5,500 mg L^−^^1^ [17] but an excess of this element in surface water can potentially threaten human health and the environment. When infants are exposed to Mn levels, a known mutagen [18], greater than those approved by the World Health Organization of 0.4 mg L^−^^1^, it might cause a high mortality risk [19]. Even though there is information concerning the level of contamination in some surface water in Mexico, little is known concerning the level of heavy metals in the water of the Conchos River.

This paper reports the results of some metal levels in waters flowing in six tributaries of the Conchos River in Chihuahua, Mexico over a two year period. To the best of our knowledge, this is the first time that a study considered the entire watershed to determine the levels of contamination. As such, these results will be useful to different authorities in analyzing potential harmful effects on human health, wildlife, the environment and the suitability of the Conchos water for beneficial utilization in general.

## 2. Materials and Methods

The Conchos River water originates in the mountains of Chihuahua, about 2,700 meters above sea level (masl). This area is located on the west side of the state and is identified as the Tarahumara region or Tarahumara mountain area. The Conchos’ stream flow then descends to the great plain with 1,000–1,500 masl, and finally the flow joins the Rio Bravo/Rio Grande water near the city of Ojinaga, Mexico. The Rio Bravo/Rio Grande serves as the natural boundary between Mexico and the United States. The most significant Conchos’ tributaries are the Florido River and the Parral River to the south, the San Pedro River in the center and the Chuviscar River which flows in central Chihuahua.

**Figure 1.**
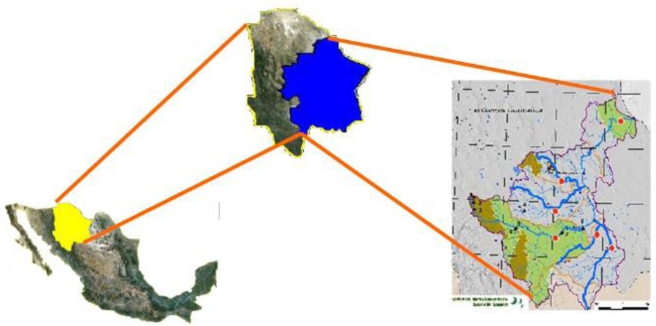
Map showing Mexico, the State of Chihuahua and the sampling location points in the Conchos River.

Six sites were selected to obtain water samples during 2005 and 2006 (Figure 1). Point 1 was located in the Chuviscar River (latitude 28°49′23.7″; longitude 105°54′57.0″; 1,279 masl) about 15 km east of the city of Chihuahua. Point 2 was located in the San Pedro River (latitude 27°57′13.2″; longitude 106°06′35.9″; 1,375 masl) approximately 5 km from the town of Satevo, before the water is being captured in the Virgenes Dam. Point 3 was sited about 2.5 km from the town of Valle de Zaragoza (latitude 27°28′15.5″; longitude 105°42′25.4″; 1,329 masl). Sampling point 4 was in the Parral River (latitude 27°40′03.4″; longitude 105°12′33.8″; 1,228 masl) about 30 km from the city of Parral. Point 5 was located in the Florido River (latitude 27°40′36.6″; longitude 105°08′37.4″; 1,225 masl) above 10 km from the city of Camargo. Sampling point 6 was situated near the city of Ojinaga (latitude 29°34′02.1″; longitude 104°26′46.1″; 786 masl) approximately 2 km above the junction with the flow from the Rio Bravo/Rio Grande.

The water samples were obtained during 2-month intervals (February, April, June, August, October and December) at each point, every year. The rainy season is very short in the north of Mexico beginning in June and end in September. The samples were collected the same day in sterilized containers, preserved in a cooler and immediately transported to the laboratory of the College of Zoo-technology and Ecology of the Autonomous University of Chihuahua, where they were placed at 4 °C for further lab analysis. Metals from the water samples were extracted according to the Mexican Norm [20] and the concentrations of As, Cr, Cu, Fe, Mn, Ni, V, Zn and Li were determined by an Inductively Coupled Plasma-Optical Emission Spectrometer (ICP-OES) model 2100 by Perkin Elmer. The water temperature, the pH and the electrical conductivity (EC) were determined *in situ* in each point. Water temperature was measured with a mercury thermometer while the pH was determined with the Oktron model 35624-50 device. The EC was calculated with a Hanna device and the units were transformed to dSm^−^^1^. Solid totals were determined following the Mexican norm [21] while total nitrogen was determined with the sum of organic nitrogen and ammonia nitrogen following the Mexican norm [22].

An analysis of variance (ANOVA) was performed for each variable to determine year, month, and location effects and their interactions. The data of the Florido River was not analyzed because this specific river was mostly dry due to activities conducted upstream and so it was particularly difficult to get water samples from this point. According to the ANOVA results, some descriptive statistics were used to visualize differences in concentration considering sampling points and location points.

## 3. Results and Discussion

**Figure 2.**
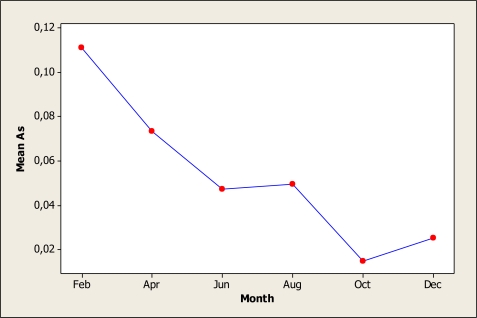
Month effect for arsenic in water samples during the period of 2005–2006.

Based on the ANOVA results, there were no significant differences in metals contents among the five sampling locations of the Conchos watershed as it was previously hypothesized which relegates the importance of point sources as contributing to these elements with respect to the others factors (months and years). Most of the differences were observed in the sampling month and in the interaction of month x year. The ANOVA for As levels detected statistical significance only for month (P = 0.008) as Figure 2 shows this main effect. The As mean for February was 0.11 mg L^−^^1^ which was the highest concentration observed while the lowest level was noted in the October samples with 0.01 mg L^−^^1^. The results presented here agree with the findings of Gutierrez *et al*. [1] who detected concentrations in the San Pedro River of Chihuahua, Mexico in a range of 0.07 to 0.16 mg L^−^^1^. Moreover, Espino-Valdez *et al*. [23] in a study carried out in central Chihuahua, Mexico with the objective of determining the level of As in well water for drinking purposes, found that 72% of the water samples exceeded the maximum limit of 0.025 mg L^−^^1^ established in the Mexican norm. These results are relevant when considering that metal concentration might be higher in groundwater than in surface water [24]. In our study, the location 1 had the maximum level of As with 0.06 mg L^−^^1^ whose results disagree with the findings of Holguin *et al*. [25] who noted a maximum level of this element as 0.035 mg L^−^^1^ in the same location during a study conducted in 2005.

Many residents established along the Conchos tributaries harvest and eat fish and other products found in this river environment. One can only assume that the inhabitants are consuming the contaminants which are present in these organisms. Even though this study did not consider a formal evaluation of fish consumption and other products, we polled the residents who live in the Conchos River area, and they confirmed that they routinely consumed fish products from the river closest to their home. If we approximate an annual consumption of 48 meals (one per week) and 400 g of wet weight in each meal, the average would be approximately 19.2 kg per person. This amount of food, if contaminated, is considered high when chronic arsenic exposure in the range of 0.01–0.04 mg kg^−^^1^d^−^^1^ is carcinogenic [6–8]. It is generally known that inorganic arsenic is the most consequential but what is not known is how much of the total arsenic is inorganic. The NRC [26] considered that 10% of the seafood is in inorganic form while other research claims this percentage is as high as 30% [27]. Recently, the USEPA [28] noted that 10% is a good percentage for freshwater fish.

Additionally, Conchos residents consume chicken and other dietary products, which may act in an additive way as they may also contain high levels of arsenic [29]. It is important to mention that there is controversy surrounding the role of ingested arsenic because some have suggested that this element should be considered more potent than before [30] while others experts questioned this statement [31]. Young adults are a special case because they may eat three to four times more food than older adults and consequently, ingest larger amounts of contaminants per unit of body mass [32]. Therefore, we highly recommend an estimate of fish consumption and the level of arsenic and other contaminants in future studies. In addition, it will be imperative to ascertain other aspects such as the use of water for cleaning dishes, bathing, washing clothes and other uses.

**Figure 3.**
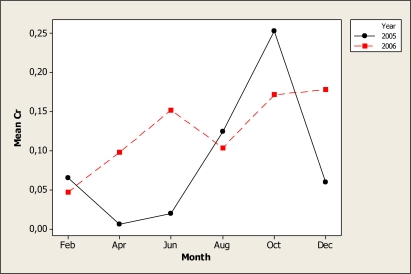
Interaction plot for Cr in water samples during two years.

The ANOVA detected differences in Cr levels as a function of sampling month (P < 0.001) and for the year-month interaction (P < 0.001). Figure 3 shows that a higher level was noted in the 2005 October samples with about 0.25 mg L^−^^1^, while the lowest concentration was observed during the April and June samplings in 2005. Location 1 gave the highest Cr value of 0.11 mg L^−^^1^, followed by location 2 and 3 with 0.10 mg L^−^^1^, while the lowest level was noted in location 4 with 0.08 mg L^−^^1^. We must point out that Cr may accumulate in freshwater fish [33] and so the fish caught in the Conchos River may be a potential health hazard for inhabitants of the area.

**Figure 4.**
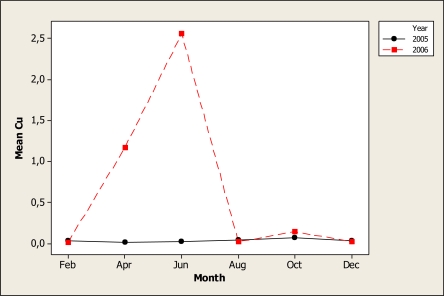
Interaction plot for Cu in water samples during two years.

The statistical analysis for Cu concentration detected significant differences for sampling year (P < 0.001), sampling month (P < 0.001) and for year-month interaction (P < 0.001) as shown in Figure 4. It is obvious that in 2005, samples were consistent when compared with 2006 samples, as April and June samples were higher than the other months tested. This spike can be explained by the fact that copper-containing fungicides are commonly used at the beginning of the year for pecan production and other crops. The concentration of Cu in the locations was in a range of 0.37 mg L^−^^1^ found in location 4 to 0.50 mg L^−^^1^ observed in location 3. This element should be tested in future studies not only on surface water but in public areas as well because it has been proven that drinking fountains may be an important source of this element [34].

**Figure 5.**
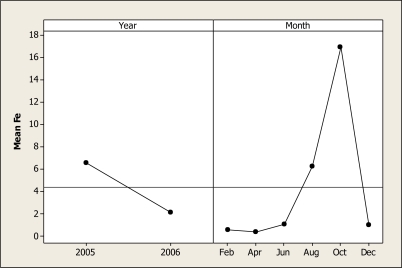
Main effects plot for Fe in water samples during two years.

The ANOVA for Fe concentration showed statistical differences as a function of year (P = 0.030) and month (P = 0.003) but no differences were noted for location or the interactions. This main effect is shown in Figure 5 where maximum Fe concentrations were noted in the October samples with approximately 16.36 mg L^−^^1^ and the August sampling with 7.0 mg L^−^^1^. With respect to year concentration, maximum levels of this element can be seen when noted in 2005 samples. It is understood that aquatic insects may suffer some toxicity at Fe concentrations of 0.320 mg L^−^^1^ and the lethal concentration in fish ranges from 0.3 to 10 mg L^−^^1^ of Fe [35,36]. The results of this study are higher than these values, meaning that the river ecosystem habitat is being negatively impacted.

**Figure 6.**
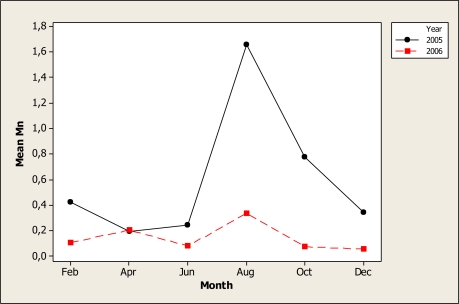
Interaction plot for Mn in water samples during two years.

The ANOVA element in regard to Mn, noted statistical differences for year (P < 0.001), month (P = 0.004) and the interaction year-month (P = 0.042) as shown in Figure 6. As evident, maximum Mn levels were noted in the August-December samples. In the location 1 the samples noted 0.56 mg L^−^^1^. In our study, a wide range of this element was observed that agreed with the findings of Schlenker *et al*. [37] who reported on water well samples values from < 0.001 mg L^−^^1^ to 0.164 mg L^−^^1^. It is interesting to point out that the Mn absorption is inversely associated with Fe levels that were discussed in the last paragraph [38]. Therefore, it is important to suggest further studies in the Conchos area that considers both elements.

**Figure 7.**
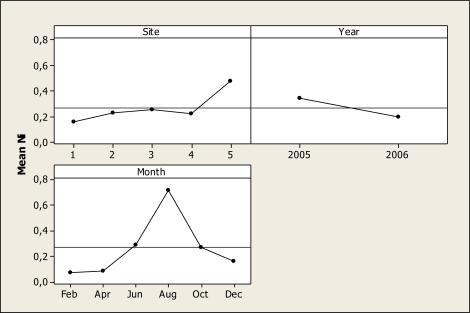
Main effects plot for Ni in water samples during the years of 2005–2006.

The ANOVA for Ni concentration showed statistical differences for sampling year (P = 0.030), sampling month (P < 0.001) and sampling location (P = 0.050) but no differences were noted for any interaction as shown in Figure 7. As shown, the maximum amount was noted in the months during and after the rainy season. Thus, in June the Ni concentration was 0.29 mg L^−^^1^, in August 0.68 mg L^−^^1^ and in December, the samples were 0.18 mg L^−^^1^. In addition, Figure 7 shows that location 5 was the most contaminated with this element reaching 0.47 mg L^−^^1^ and that the water tested in 2005 contained more Ni than the 2006 samples. The results of this study concerning Ni levels are higher than those reported by Holguin *et al*. [25] who found levels of approximately 0.07 mg L^−^^1^ in the Conchos River near the city of Ojinaga. Moreover, we must point out that in all locations the level of this element was higher than the Mexican standards for irrigation water established in 0.2 mg L^−^^1^. This element is considered a potential human carcinogen [39] as the World Health Organization has established the drinking water guideline in 0.02 mg L^−^^1^.

**Figure 8.**
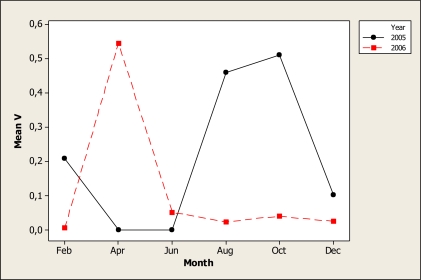
Interaction plot for V in water samples during two years.

The V concentration was statistically different for year (P < 0.001), month (P < 0.001) and for the interaction year × month (P < 0.001) but no differences were discovered for location and the other interactions. Figure 8 shows that the highest level of this element was noted in 2005 during the April sampling and in 2006 during the August and October samples. The mean concentration for location was similar, in a range of 0.14 mg L^−^^1^ in the location 2 to 0.17 mg L^−^^1^ in the location 1. V is located mostly in the kidneys, lungs and bones but the total amount of this element in the human body is estimated to be less than 1 milligram. Even though V is considered as an essential element [40], its specific function in the human metabolism is uncertain.

**Figure 9.**
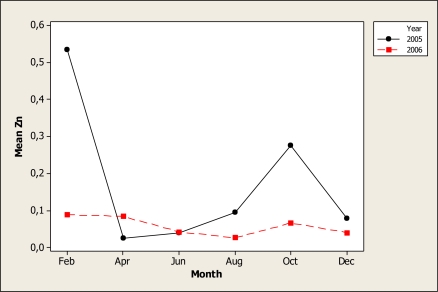
Interaction plot for Zn in water samples during two years.

For Zn, the ANOVA detected significant differences in year (P < 0.001), month (P < 0.001) and the interaction year X month (P < 0.001). Figure 9 shows a consistent concentration during both years, with the exception of water samples collected during February 2005, when a peak occurred and a sharper peak again in the October samples. The mean of Zn in the locations varied from 0.08 mg L^−^^1^ in location 4 to 0.15 mg L^−^^1^ in location 1 samples. At this particular point, the level was higher than the threshold level recommended for aquatic organisms of 0.12 mg L^−^^1^ [41]. We must point out that Zn is considered an essential element for aquatic organisms [42], but it can be toxic to aquatic life in high concentrations [43] and can damage the pancreas and kidneys in humans [44].

**Figure 10.**
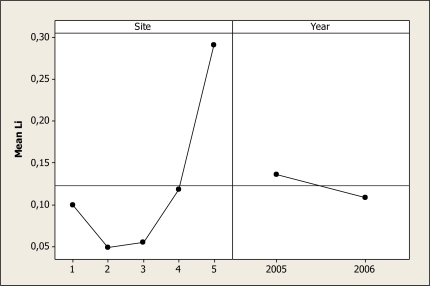
Main effects plot for Li in water samples during two years.

Lithium concentration was different as a function of year (P = 0.037) and location (P = 0.028) and the maximum level of this element was found in locations 1 and 5 (Figure 10). In addition, it was noticed that the Li concentration was higher in the 2005 samples. In another study carried out in the Conchos near Ojinaga, Holguin *et al*. [25] found levels of Li similar to the results reported here. These researchers noted levels of Li in a range of 0.06 mg L^−^^1^ in the June sample and 0.13 mg L^−^^1^ in the April samples. Our results showed a Li peak in December with concentration as high as 0.28 mg L^−^^1^ which concurs with the results reported by Gutierrez *et al*. [1] of 0.33 mg L^−^^1^ in water sampled from other tributaries of the Conchos River. This element may be a major ecological risk in the water of the Conchos River when considering that some levels are higher than 0.04 mg L^−^^1^ and may be toxic to some aquatic insect larvae [45].

As to water temperature, the ANOVA detected significances only as a function of sampling month (P < 0.001). As expected, low records were noted in the February samples with 14 °C with increases the following months, reaching 26 °C in the August samples to a low again in the December samples with 21 °C. The pH values were different for year (P = 0.006), month (P < 0.001), location (P = 0.013) and the interaction year × month (P = 0.018). The lowest pH level was in the January samples with 7.2 and the highest level was detected in the June samples with 8.3. Considering location, the highest level was noted in location 2 with 7.7 and the lowest was observed in location 1 samples with 6.9. The EC was different only for location (P < 0.001) observing the highest amount in location 1 with 1.65 dSm^−^^1^ and the lowest in location 3 samples with 0.38 dSm^−^^1^. Total N was different for year (P = 0.018), month. (P = 0.018), location (P < 0.001) and the interaction month x location (P < 0.001). A higher N level was noted in the February samples with 7.12 mg, while the lowest amount was observed in the August samples with 0.24 mg. In the Conchos River near Ojinaga (location 5), the highest level of total N was measured with 3.77 mg while the lowest level was noted in location 4 with 1.66 mg.

## 4. Conclusions

We have identified elements that represent a potentially significant public health challenge that requires urgent attention from different government agencies and future research involving human health. In Mexico, it will be important to have the water of this watershed free from harmful contaminants and this study represents the first step of this project. In general, we did not find differences in metal concentrations among the five locations in the Conchos watershed, suggesting that no apparent point source was located. Therefore, one can assume that the presence of metals and physical and chemical characteristics of the water must be related mostly to surface runoff. As it was expected downstream locations such as Ojinaga had a higher metal concentration in water than most upstream locations like Zaragoza and Satevo. We recommend a monitoring program of the chemical contamination of the Conchos watershed with special emphasis on the recreational harvesting of fish in the area and knowing the ecological risks involved.
